# Bilingualism Enhances Metalinguistic Awareness in Autism: Extending the Two‐Dimensional Grammaticality Judgment Task

**DOI:** 10.1002/aur.70173

**Published:** 2025-12-29

**Authors:** Pauline Wolfer, Franziska Baumeister, Stephanie Durrleman

**Affiliations:** ^1^ Autism, Bilingualism, Cognitive and Communicative Development Research Group (ABCCD) Faculty of Science and Medicine, University of Fribourg Fribourg Switzerland

**Keywords:** autism spectrum disorder, bilingualism, grammaticality judgment task, metalinguistic awareness

## Abstract

Bilingualism has been associated with enhanced metalinguistic awareness (MA), the ability to reflect upon language. However, findings remain mixed, and little is known about how proficiency in the most proficient (L1) and second‐best language (L2) contribute to MA, especially in children with autism spectrum disorder (ASD), who often present heterogeneous cognitive and linguistic profiles. We tested 270 children aged 5–12 (90 autistic, 180 neurotypical) using a two‐dimensional Grammaticality Judgment Task (GJT) requiring two components of MA: *analyzed knowledge* (understanding of grammatical rules) and *cognitive control* (suppressing irrelevant semantic cues). Bilingualism was examined both categorically (monolingual vs. bilingual) and continuously (L2 proficiency), using generalized linear mixed‐effects models controlling for age, nonverbal IQ, and L1 proficiency. Among neurotypical children, no significant bilingual advantage was found. In contrast, bilingual autistic children significantly outperformed monolingual peers on items requiring cognitive control, and higher L2 proficiency was associated with better overall GJT performance. These findings advance understanding of how bilingualism relates to metalinguistic abilities in autism and suggest that it is not only non‐detrimental but may confer specific metalinguistic benefits. The study underscores the importance of combining categorical and continuous approaches to bilingualism to better capture individual variability in neurodiverse populations.

## Summary

Bilingual autistic children did better than monolingual autistic children at focusing on language and ignoring distractions. Stronger skills in a second language were linked to better overall performance for autistic children. Learning more than one language can support autistic children's language thinking skills and should be seen as a strength.

## 1 Introduction

Metalinguistic awareness (MA), the ability to reflect on language as an object of thought (Tunmer and Herriman 1984), is central to linguistic and cognitive development (Pratt and Grieve 1984). In practical terms, MA notably enables children to separate *form* from *meaning*: they can judge that “Colorless green ideas sleep furiously” (Chomsky 1957) is grammatically well‐formed despite being nonsensical, or that “He goed to school” is meaningful but ungrammatical. Beyond structural judgments, MA also supports everyday interactions that require interpreting meaning beyond the literal, such as understanding metaphors like “Time is a thief.” These skills allow speakers to monitor and repair communication, adjust language to different listeners, and infer meanings from context (Patterson 2011). Developing explicit control over language fosters literacy, reading comprehension, and flexible language use across settings (Melogno et al. 2022; Nippold 2004; Patterson 2011).

Bilingual neurotypical (NT) children often outperform monolingual peers on metalinguistic tasks, such as grammaticality judgment (Adesope et al. 2010), likely due to managing multiple language systems, which can enhance sensitivity to linguistic structure and promote a more analytical approach to language (Bialystok 1988; Galambos and Goldin‐Meadow 1990). Whether similar effects occur in autistic children remains unclear, though findings could inform educational and clinical practices. Evidence shows that morphological, phonological, and orthographic awareness each make unique contributions to reading comprehension and literacy development in neurotypical children (Li and Wu 2015; Lyster et al. 2021; Melby‐Lervåg et al. 2012). Interventions that explicitly target metalinguistic awareness, for instance by engaging children in analyzing word structure, resolving linguistic ambiguity, or reflecting on grammatical form, have been found to improve reading fluency (Siu et al. 2018). In autistic children, linguistic awareness skills predict reading and spelling abilities, suggesting that interventions designed to strengthen explicit reflection on language form may improve literacy and communicative repair strategies (Henbest and Apel 2024).

### 1.1 A Two‐Dimensional Framework to Examine Metalinguistic Awareness

Bialystok and Ryan (1985) proposed a two‐dimensional framework specifying two dimensions involved in metalinguistic tasks: *analyzed knowledge* (the explicit understanding and knowledge of linguistic features) and *cognitive control* (the ability to focus attention on these features and manipulate them). This framework has been widely applied in comparisons of monolingual and bilingual children aged 5–10 (Bialystok 1986, 1988; Bialystok and Barac 2012; Bialystok and Majumder 1998; Cromdal 1999), often through an adapted Grammaticality Judgment Task (GJT). In these tasks, children judged the grammaticality of spoken sentences, some of which included semantic anomalies. Four sentence types were designed to vary demands on the two dimensions:

1. *GM, Grammatically Correct and Meaningful*: easiest sentences, as they are congruent (i.e., both grammar and meaning are correct), and no error must be detected.
2. *gm, Grammatically Incorrect With a Semantic Anomaly*: harder than GM, as detecting grammatical mistakes is more difficult than identifying grammatically correct sentences (Hakes 1980).
3. *Gm, Grammatically Correct But Semantically Anomalous*: requires greater *cognitive control* to inhibit (misleading) meaning, which is processed automatically (Hakes 1980).
4. *gM, Grammatically Incorrect But Meaningful*: requires greater *analyzed knowledge* to detect errors without the interference of misleading meaning.

Individual differences in performance often reflect variability in language proficiency in the testing language, as the ability to detect grammatical or semantic violations depends on children's command of the language in which the task is administered. In bilingual children, stronger skills in the testing language support more accurate sentence analysis (Bialystok and Barac 2012). At the same time, proficiency in the second language (L2) can also impact metalinguistic awareness. According to the *Interdependence Hypothesis* (Cummins 1991), underlying linguistic knowledge can transfer across languages, such that exposure to two language systems may enhance grammatical understanding (Bialystok 1988; Verhoeven 2007). Together, these factors help explain variability in MA, especially in populations with diverse linguistic profiles.

### 1.2 Bilingualism Effects on GJT

#### 1.2.1 In Neurotypical (NT) Children

Findings from the two‐dimensional paradigm suggest that bilingualism effects on MA depend both on the dimension assessed (*analyzed knowledge* vs. *cognitive control*) and on children's proficiency in their second best‐language (L2).

For the *cognitive control* dimension (i.e., performance on Gm sentences), bilingual children often outperformed monolinguals (Bialystok 1986, 1988; Bialystok and Majumder 1998; Cromdal 1999). Some studies find this advantage regardless of L2 proficiency (Bialystok and Majumder 1998), while others report it only among bilinguals with high L2 proficiency (Cromdal 1999). Bilinguals typically score higher on Gm (high *control*) than gM (high *analyzed knowledge*) items, whereas monolinguals show the opposite pattern, likely reflecting different processing strategies (i.e., prioritize the processing of meaning) and reduced ability to inhibit semantic interference (Barac and Bialystok 2012; Bialystok 1986).

For *analyzed knowledge* (gM sentences), results are mixed. Some studies report no group differences between bilingual and monolingual children (Bialystok 1986; Bialystok and Majumder 1998), while others find that bilingual advantages emerge only among children with high L2 proficiency (Bialystok 1988; Cromdal 1999). This suggests a possible L2 proficiency threshold for gains in *analyzed knowledge*, though the minimum level remains unclear

Most prior research has treated L2 proficiency categorically (“monolingual,” “*partially* bilingual,” or “*fully* bilingual”), often based on receptive vocabulary scores (Bialystok 1988; Cromdal 1999). Such an approach may obscure meaningful interindividual variability. Methodological inconsistencies further limit interpretation: some studies tested subgroups of children in their weaker language (Bialystok and Majumder 1998), combined results obtained in L1 and L2 (Bialystok 1988), or found effects only when children were tested in their weaker language (Cromdal 1999). These variations complicate efforts to isolate the role of L2 proficiency in tasks administered in the most proficient language (L1).

Recent recommendations call for combining continuous and categorical measures, with theory‐driven designs reflecting individual variability (De Houwer 2023; Kremin and Byers‐Heinlein 2021). Such approaches are particularly relevant in autism research, where cognitive and linguistic profiles vary widely (Prévost and Tuller 2022).

#### 1.2.2 In Autistic Children

MA has been extensively studied in NT populations, but remains poorly understood in Autism Spectrum Disorder (ASD). Autistic children show diverse cognitive and linguistic profiles (American Psychiatric Association 2022), often including challenges in structural language (Rapin and Dunn 2003; Schaeffer et al. 2023; Silleresi 2023) and executive function (Craig et al. 2016; Leung et al. 2016). Longitudinal and cross‐sectional studies indicate that between one‐third and one‐half of school‐aged autistic children meet criteria for structural language impairment, while others display age‐appropriate or even advanced language skills (Pickles et al. 2009; Rapin and Dunn 2003; Schaeffer et al. 2023). This heterogeneity has important implications for metalinguistic performance, as tasks requiring reflection on linguistic form partly depend on grammatical and lexical competence. Accounting for such variability is therefore essential when comparing autistic and neurotypical groups on MA tasks.

Regarding MA, a preliminary study found that autistic children (9–17 years) performed below NT peers on tasks assessing metalinguistic competence, including inferential language understanding and ambiguity resolution (Lewis et al. 2007). However, a recent two‐dimensional GJT with nonverbal responses found no differences between autistic and NT school‐aged, suggesting that autism‐related metalinguistic difficulties may lessen when language complexity is reduced (Wolfer et al. 2024).

Studying bilingualism in both autistic and NT children can clarify whether observed findings generalize across populations with differing cognitive and linguistic profiles. This aligns with calls to treat bilingualism as a heterogeneous, individualized experience (de Bruin 2019; de Bruin and Hayiou‐Thomas 2022). It also addresses a pressing societal concern, as many parents worry that dual‐language exposure may exacerbate the linguistic, cognitive, and communicative challenges associated with autism (Drysdale et al. 2015; Howard et al. 2021; Yu 2013).

### 1.3 The Present Study

#### 1.3.1 Purpose

This study examined how bilingualism and second‐language (L2) proficiency relate to GJT performance in school‐aged autistic and neurotypical children. Adopting a two‐dimensional cognitive framework previously applied only to NT populations (e.g., Bialystok 1986, 1988; Bialystok and Majumder 1998; Cromdal 1999), we aimed to: (1) replicate reported bilingualism effects in NT children aged 5–12; (2) assess the direct impact of L2 proficiency on MA; and (3) determine whether these effects generalize to age‐matched peers with ASD. The 5–12 age range was selected to encompass the developmental period in which metalinguistic skills typically emerge and consolidate (Melogno et al. 2022).

#### 1.3.2 Research Questions

*Do NT and autistic bilinguals outperform monolinguals peers in MA requiring cognitive control (Gm sentences)?*

Prior research indicates that NT bilinguals tend to outperform monolinguals on such items (Bialystok 1986, 1988; Bialystok and Majumder 1998).

*How does L2 proficiency impact performance on MA requiring cognitive control (Gm sentences) in NT and autistic children?*

Following current recommendations of bilingual operationalization (De Houwer 2023; Kremin and Byers‐Heinlein 2021; Rothman et al. 2023), we assessed the direct impact of L2 proficiency. Based on Cromdal (1999), we predicted higher L2 proficiency would be associated with better Gm performance.

*How does L2 proficiency impact performance in MA requiring analyzed knowledge (gM sentences) in NT and autistic children?*

In NT children, we predicted a positive effect of L2 proficiency on gM sentences (Bialystok 1988), although an absence of effect is also plausible (Bialystok 1986; Bialystok and Majumder 1998).

This is the first study to investigate bilingualism's impact on MA in autistic children using this framework. Given its exploratory nature, no specific predictions were made for the autistic group. However, we anticipated greater performance variability due to known heterogeneity in nonverbal IQ (Silleresi 2023), executive functioning (Craig et al. 2016; Demetriou et al. 2019), and language skills (Schaeffer et al. 2023), all factors likely to influence performance in both MA dimensions (Bialystok and Barac 2012).

## 2 Method

### 2.1 Participants

#### 2.1.1 Groups

Participants were prospectively recruited between January 2023 and August 2024 through contacts with primary schools, autism associations, after‐care providers, psychologists, speech‐language therapists, and the online recruitment platform *BuildClinical* (US). All caregivers provided written informed consent, and participants were compensated with a gift card (CHF 35, 35 €, 60 CAD, or 35 USD).

The data of *N* = 270 children between 5 and 12 years old were analyzed; *N* = 90 autistic children (9;1 ± 1;9 years; 84.4% male), and *N* = 180 NT children (8;6 ± 1;11 years; 48.9% male). Autistic participants had an official diagnosis of autism spectrum disorder established by a certified professional prior to study inclusion with standardized tools (e.g., *Autism Diagnosis Observation Schedule 2nd Edition*, *ADOS‐2;* Lord et al. 2012; *Autism Diagnosis Interview‐Revised, ADI‐R*; Lord et al. 1994). Neurotypical children had no diagnosis or suspicion of neurodevelopmental disorder of any kind. All caregivers filled in the *Social Communication Questionnaire* (SCQ; Rutter et al. 2003), a 40‐item parental questionnaire that screens for autistic traits. Neurotypical children all scored below the cut‐off of 15 and presented, as expected, a lower average score than their autistic peers (*W* = 14,924, *p* < 0.001). Sample characteristics are presented in Table 1.

**TABLE 1:** Characteristics of the sample.

	Autistic children	Neurotypical children
	(*N* = 90)	(*N* = 180)
Age (months)		
Mean (SD)	109 (21.7)	102 (23.7)
Median [Min, Max]	112 [61, 143]	103 [61, 143]
Sex assigned at birth		
Female	14 (15.6%)	92 (51.1%)
Male	76 (84.4%)	88 (48.9%)
SCQ score		
Mean (SD)	19.5 (7.07)	4.06 (2.99)
Median [Min, Max]	20 [3, 34]	4 [0, 14]
Parental educational level (Min 1 to Max 5)		
Mean (SD)	4.00 (1.11)	4.60 (0.70)
Median [Min, Max]	4 [1, 5]	5 [2, 5]
Language of testing, L1 *(N; proportion of the sample)*		
English	9 (10.1%)	38 (21.1%)
French	37 (41.1%)	41 (22.8%)
German	19 (21.1%)	66 (36.7%)
Italian	3 (3.3%)	18 (10.0%)
Spanish	22 (24.4%)	17 (9.4%)

*Note*: Missing data: SCQ—5 autistic, 1 NT; Parental educational level corresponds to the highest level of education achieved by the caregivers, from (1) elementary school [ASD: 3.3%; NT: 0.0%], (2) middle school [ASD: 6.7%; NT: 2.2%], (3) high school [ASD: 21.1%; NT: 6.7%], (4) post‐secondary degree [ASD: 24.4%; NT: 13.3%], to (5) university [ASD: 44.4%; NT: 77.8%].

#### 2.1.2 Language of Testing

All participants were tested in their L1, identified by caregivers as the child's most proficient language and corresponding to the societal language of the testing site. Testing took place in English in the United States and the United Kingdom, in French in France and French‐speaking Canada, in German in Germany, in Spanish in Spain, and in French, German, or Italian in Switzerland.

The linguistic environments represented in the sample reflect a range of monolingual and multilingual contexts typical of children growing up in Western educational systems. Participants tested in France, Germany, the United Kingdom, and the United States were drawn from predominantly monolingual environments, where the language of testing is also the main societal and instructional language. In contrast, children tested in Canada were recruited in Montreal, a bilingual city where both French and English have official status; however, all Canadian participants were educated primarily in French, and French was their most proficient and home language. Participants tested in Spain were recruited in Catalonia, a region where both Spanish and Catalan are widely used and children typically acquire proficiency in both languages through schooling and daily exposure. Finally, participants tested in Switzerland were drawn from French‐, German‐, or Italian‐speaking cantons of a highly multilingual country, where societal bilingualism is common and some regions are officially bilingual. This diversity in linguistic environments reflects the real‐world variability of bilingual experience.

#### 2.1.3 Bilingualism Operationalization

Information on children's linguistic background was obtained using the *Quantifying Bilingual Experience* questionnaire (Q‐BEx; De Cat et al. 2022). Caregivers provided details on up to three languages, including exposure, use, and estimated proficiency.

For RQ1, participants were classified as “bilinguals” or “monolinguals.” Children were considered *bilinguals* if (a) parents reported exposure to a second language, and (b) the child was estimated by caregivers to have at least some proficiency in *understanding*, *speaking*, *writing*, or *reading* it. This classification yielded four groups: bilingual children with ASD (*N* = 66), monolingual children with ASD (*N* = 24), bilingual NT children (*N* = 121), monolingual NT children (*N* = 59).

For RQ2 and RQ3, L2 proficiency was indexed using caregivers' estimates of the child's proficiency in their second‐best language. Full details of bilingual characteristics and L2 proficiency calculation are provided in Appendix S1.

### 2.2 Measures

All participants were tested individually in a quiet room by a trained experimenter, with breaks provided as needed. The assessment was fully screen‐based and relied on nonverbal responses, minimizing verbal demands and ensuring standardized instructions across participants and language versions. Testing was conducted by trained experimenters affiliated with collaborating research teams in Switzerland, France, Germany, Spain, the United States, the United Kingdom, and Canada. Each site had a designated local coordinator responsible for participant recruitment and compliance with the study protocol. Standardization was maintained through centralized online and in‐person onboarding sessions, detailed protocol manuals, and regular supervision meetings led by the coordinating lab, ensuring uniform administration across sites. Data and analysis code are available at: https://osf.io/kbyc6/

#### 2.2.1 Grammaticality Judgment Task (GJT)

We used the same two‐dimensional GJT as in Wolfer et al. (2024), based on Bialystok (1986). Children judged pre‐recorded sentences for grammatical well‐formedness by “giving” an on‐screen character wither a piece of candy (correct) or a sock (incorrect). The task included 32 sentences (examples in Appendix S2) divided into four types with reduced linguistic difficulty:

1. *GM Sentences*: Grammatically correct, semantically appropriate;
2. *gm Sentences*: Grammatically incorrect, semantically anomalous;
3. *Gm Sentences*: Grammatically correct, semantically anomalous (greater demand on *cognitive control*);
4. *gM Sentences*: Grammatically incorrect, semantically appropriate (greater demand on *analyzed knowledge*).

Grammatical violations involved determiner–noun reversals and subject–verb disagreement; semantic anomalies resulted from implausible agent‐action pairings. Sentences were short and structurally simple to ensure accessibility. Each correct response scored 1 point (max total = 32; max per sentence type = 8). Most participants completed the full task; exceptions were three autistic children (28–31 items) and two NT children (31 items). Further task details appear in Appendix S2 and Wolfer et al. (2024).

#### 2.2.2 Receptive Vocabulary

Receptive vocabulary in the participant's L1 was measured using the *Peabody Picture Vocabulary Test* (PPVT‐4; Dunn and Dunn 2007) or its adapted version in the testing language (Appendix S3). Participants listened to pre‐recorded words and selected the matching picture among four options. *Z*‐scores were calculated from the normative data for each language version to ensure comparability across participants, ages, and language versions.

In our sample, 32.9% of autistic participants scored below ≤ −1.25 SD on the PPVT, compared to 3.8% among neurotypical peers (ASD: 29.4% ≤ −1.50 SD, NT: 3.1%), a common threshold to identify language impairment (Nudel et al. 2023). These proportions fall within the range reported in large‐scale studies (Nudel et al. 2023; Pickles et al. 2009; Schaeffer et al. 2023).

#### 2.2.3 Nonverbal IQ

Nonverbal IQ was assessed with the *Raven's‐2* (Raven et al. 2018), in which participants selected the missing piece completing a visual pattern from five choices. This measure is widely considered an index of fluid intelligence. Standardized IQ scores were used in the analyses.

Table 2 presents standardized scores for receptive vocabulary and non‐verbal reasoning, which were, on average, lower in the autistic group than in the neurotypical group (Receptive vocabulary: *W* = 3615.5, *p* < 0.001; Nonverbal IQ: *W* = 5492, *p* = 0.00018). Both variables were entered as covariates in all statistical models to control for individual differences in language ability and nonverbal reasoning.

**TABLE 2:** Receptive vocabulary and nonverbal IQ scores by group.

	Autistic children	Neurotypical children
	(*N* = 90)	(*N* = 180)
Receptive vocabulary (PPVT, *z*‐score)		
Mean (SD)	−0.57 (1.67)	0.67 (1.11)
Median [Min, Max]	−0.60 [−4.00, 3.27]	0.64 [−2.87, 4.00]
Nonverbal IQ (Raven's 2, IQ score)		
Mean (SD)	95.4 (14.6)	102 (12.1)
Median [Min, Max]	93 [69, 131]	103 [71, 133]

*Note*: Missing data: PPVT—5 autistic, 20 NT; Nonverbal IQ—1 autistic, 8 NT.

### 2.3 Analysis Plan

Generalized linear mixed‐effects models were fitted separately for each research question using a logit link. The dependent variable was item‐level task accuracy (0 or 1). Two planned sentence‐type contrasts indexed the *cognitive control* (Gm vs. GM) and *analyzed knowledge* (gM vs. GM) dimensions of the GJT.

For RQ1, we examined the interaction between diagnostic group (ASD/NT) and bilingual status (monolingual/bilingual). For RQ2 and RQ3, models included *L2 proficiency* as a continuous predictor. All models controlled for *age*, *language version*, *receptive vocabulary*, and *nonverbal IQ*. *Participants* and *items* were entered as random intercepts. Full modeling specifications are provided in Appendix S4.

## 3 Results

Both observed (descriptive) and predicted accuracy values are reported below. Observed accuracy refers to groups' raw accuracy scores, whereas predicted accuracy corresponds to the model‐based estimates derived from the generalized linear mixed‐effects regression analyses, which adjust for covariates (e.g., *age*, *language version*, *non‐verbal IQ*, *receptive vocabulary*) and random effects.

### 3.1 Descriptive Performance

**FIGURE 1:**
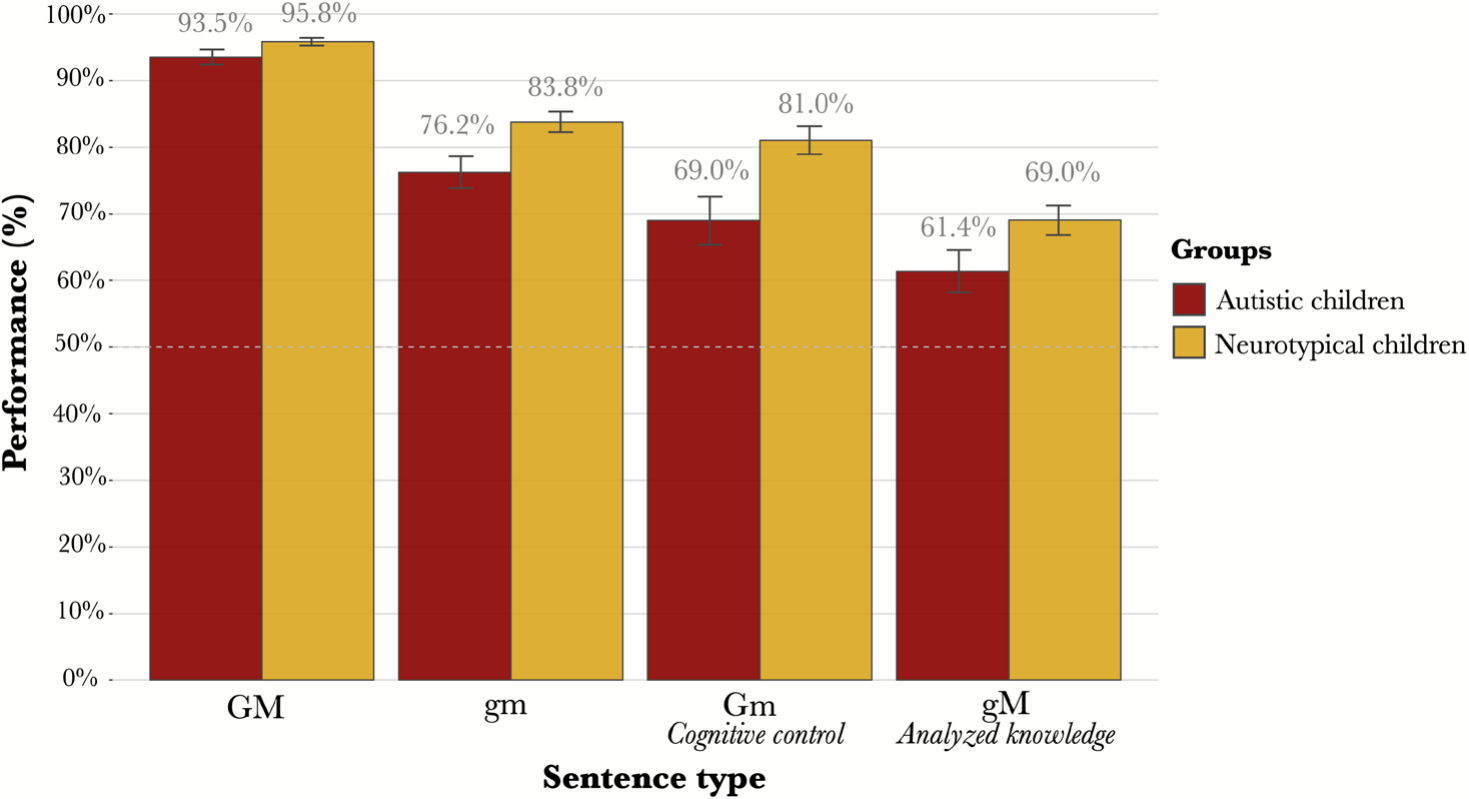
Observed mean accuracy scores (%) across participants, by group and sentence type. The gray dotted line represents the chance‐level accuracy (50%). Error bars indicate the SEM.

Both groups performed well overall: children with ASD achieved a mean accuracy of 75.0% (±1.5 Standard Error of the Mean; SEM) across all sentence types, while NT children reached 82.4% (±1.5 SEM). Both groups showed the same performance hierarchy (Figure 1), with highest accuracy on GM sentences, followed by gm, then Gm, and lowest on gM.

### 3.2 Generalized Mixed Effects Models

#### 3.2.1 RQ1: Bilinguals Versus Monolinguals Comparison on MA Requiring Cognitive Control

The model revealed a significant three‐way interaction between *group*, *bilingual status*, and *sentence type Gm relative to GM* (comparison [a]; Estimate = 0.825, SE = 0.249, *z* = 3.314, *p* < 0.001; Table 3).

**TABLE 3:** Output of the GLMM model for RQ1.

	Estimate	Standard error	*z* value	*p*
(Intercept)	1.794	0.131	13.673	< 0.001
Group	−0.676	0.186	−3.638	< 0.001
Bilingual status	0.348	0.170	2.052	0.040
Gm versus GM[a]	−0.539	0.172	−3.139	0.002
gM versus GM[b]	−1.111	0.170	−6.525	< 0.001
Sentence type [omnibus]	0.015	0.210	0.074	0.941
Age	0.839	0.074	11.375	< 0.001
Nonverbal IQ	0.180	0.077	2.332	0.020
Receptive vocabulary in L1 (PPVT)	0.317	0.056	5.625	< 0.001
Language_English	−2.675	0.730	−3.664	< 0.001
Language_French	2.827	0.644	4.389	< 0.001
Language_German	1.764	0.674	2.616	0.009
Language_Italian	−0.807	0.980	−0.823	0.410
Group: Bilingual status	0.623	0.321	1.943	0.052
Group: Gm versus GM[a]	−0.337	0.124	−2.710	0.007
Group: gM versus GM[b]	0.445	0.124	3.576	< 0.001
Group: Sentence type[omnibus]	−0.165	0.150	−1.103	0.270
Bilingual status: Gm versus GM[a]	0.110	0.125	0.880	0.379
Bilingual status: Gm versus GM[b]	0.009	0.124	0.070	0.944
Bilingual status: Sentence type[omnibus]	0.022	0.150	0.146	0.884
Group: Bilingual status: Gm versus GM[a]	0.825	0.249	3.314	< 0.001
Group: Bilingual status: gM versus GM[b]	−1.091	0.249	−4.387	< 0.001
Group: Bilingual status: Sentence type[omnibus]	0.571	0.299	1.910	0.056

*Note*: [a]: planned comparison [a] between Gm and GM sentences; [b]: planned comparison [b] between gM and GM sentences; [omnibus] corresponds to the overall effect of sentence type.

To identify the source of the interaction, follow‐up analyses were conducted separately in each group. In NT children, the two‐way interaction between *bilingual status* and *comparison [a]* was not significant (*p* = 0.068; Table A, Appendix S5), indicating no difference between monolingual and bilingual NT children. In contrast, in autistic children, this interaction was significant (Estimate = 0.533, SE = 0.187, *z* = 2.854, *p* = 0.004; Table B in S5), with bilingual autistic children outperforming monolingual peers on both Gm sentences (Estimate = 1.755, SE = 0.638, *z* = 2.275, *p* = 0.006; Table C in S5) and baseline GM sentences (Estimate = 1.195, SE = 0.491, *z* = 2.435, *p* = 0.015; Table D in S5). The predicted accuracy for Gm sentences was 80.5% (95% CI: 71.0–87.5) in monolingual autistic children and 53.7% (95% CI: 38.0–68.7) in bilingual autistic children.

Among bilinguals, there was no significant effect of *group* or interaction with *sentence type*, indicating comparable performance between bilingual autistic and NT children (Table E in S5). In monolinguals, however, a significant interaction emerged (Estimate = 0.983, SE = 0.211, *z* = −3.644, *p* < 0.001; Table F in S5), with NT children outperforming autistic peers on Gm sentences (Estimate = −3.126, SE = 1.039, *z* = −3.010, *p* = 0.003; Table G in S5).

**FIGURE 2:**
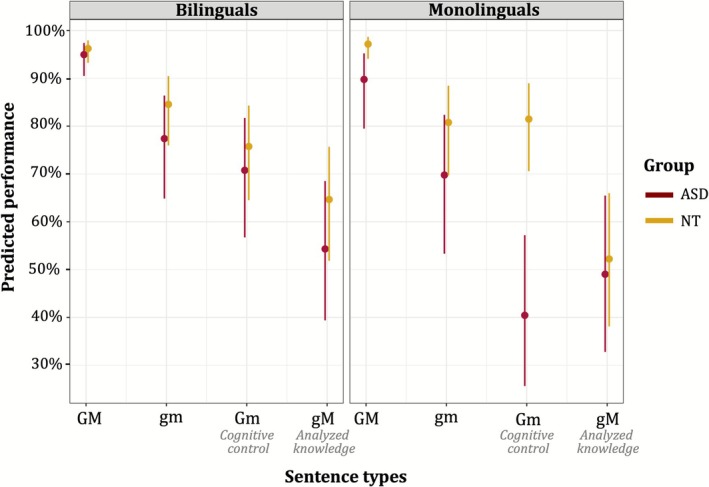
Predicted performance in the four sentence types in bilingual and monolingual autistic and NT children.

Figure 2 displays predicted performance by *sentence type*, *group*, and *bilingual status*.

#### 3.2.2 RQ2: Effect of L2 Proficiency on MA Requiring Cognitive Control

**FIGURE 3:**
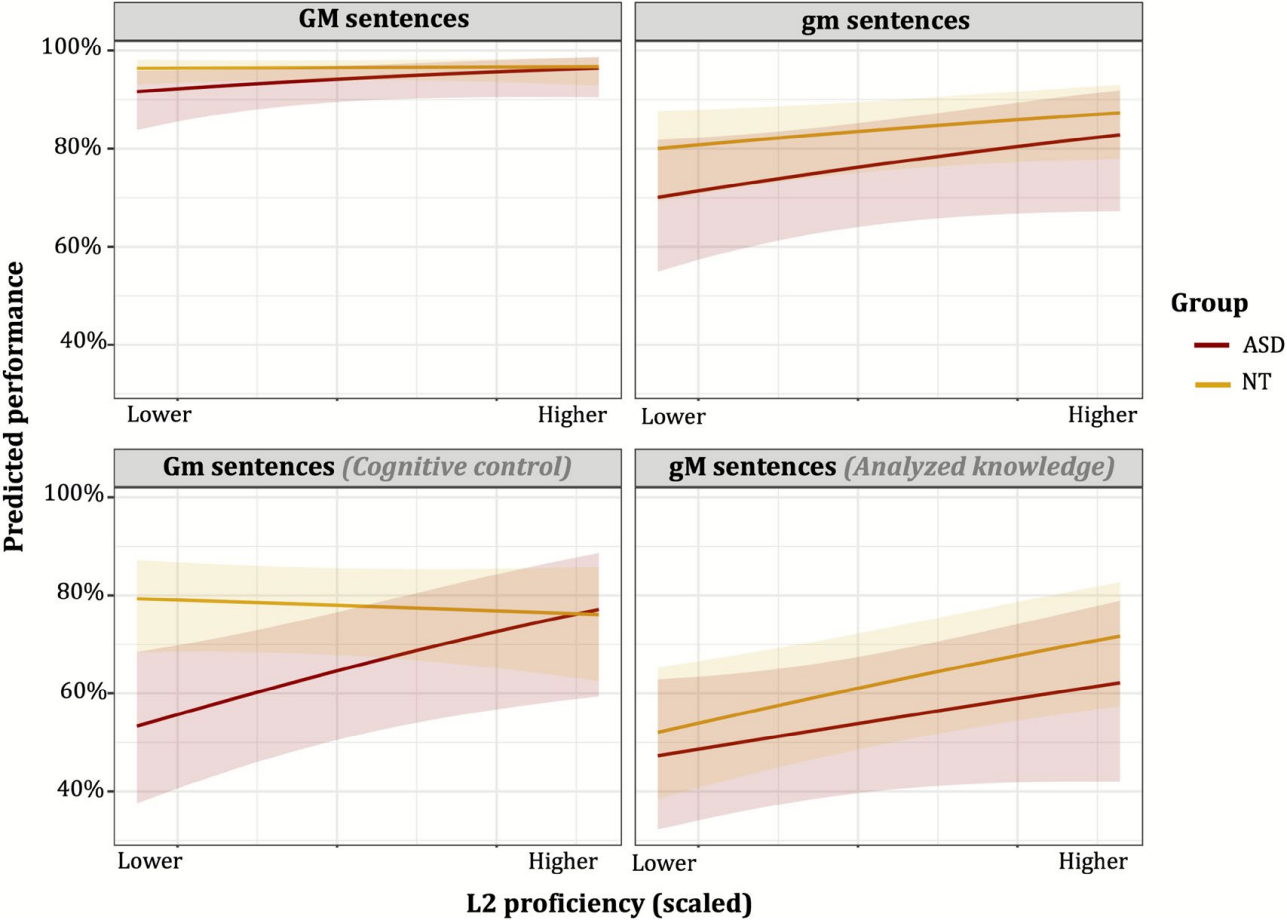
Predicted performance on each sentence type in autistic and NT children as a function of L2 proficiency.

Figure 3 shows predicted probabilities for L2 proficiency on metalinguistic performance.

No significant two‐ or three‐way interactions involving *L2 proficiency* and *comparison [a]* (Gm vs. GM) were found (Table 4), indicating that L2 proficiency did not differentially affect NT and autistic children on Gm sentences. However, L2 proficiency significantly predicted overall task performance across groups (Estimate = 0.199, SE = 0.089, *z* = 2.236, *p* = 0.025; Table 4), with both autistic and NT children with higher L2 proficiency outperforming those with lower proficiency.

**TABLE 4:** Output of the GLMM model for RQ2.

	Estimate	Standard error	*z* value	*p*
(Intercept)	1.890	0.128	14.756	< 0.001
Group	−0.491	0.178	−2.755	0.006
L2 proficiency	0.199	0.089	2.236	0.025
Gm versus GM[a]	−0.497	0.169	−2.938	0.003
gM versus GM[b]	−1.128	0.168	−6.707	< 0.001
Sentence type[omnibus]	0.032	0.207	0.153	0.878
Age	0.812	0.074	10.964	< 0.001
Nonverbal IQ	0.170	0.078	2.187	0.029
Receptive vocabulary in L1 (PPVT)	0.302	0.056	5.388	< 0.001
Language_English	−2.612	0.739	−3.536	< 0.001
Language_French	2.849	0.651	4.376	< 0.001
Language_German	1.852	0.676	2.740	0.006
Language_Italian	−0.544	0.988	−0.551	0.582
Group: L2 proficiency	0.172	0.155	1.108	0.268
Group: Gm versus GM[a]	−0.159	0.114	−1.389	0.165
Group: gM versus GM[b]	0.207	0.114	1.820	0.069
Group: Sentence type[omnibus]	−0.039	0.137	−0.281	0.779
L2 proficiency: Gm versus GM[a]	−0.038	0.060	−0.634	0.526
L2 proficiency: Gm versus GM[b]	0.058	0.060	0.971	0.332
L2 proficiency: Sentence type [omnibus]	−0.022	0.072	−0.302	0.763
Group: L2 proficiency: Gm versus GM[a]	0.229	0.119	1.923	0.054
Group: L2 proficiency: gM versus GM[b]	−0.292	0.119	−2.459	0.014
Group: L2 proficiency: Sentence type[omnibus]	0.126	0.144	0.875	0.382

*Note*: [a]: planned comparison [a] between Gm and GM sentences; [b]: planned comparison [b] between gM and GM sentences; [omnibus] corresponds to the overall effect of sentence type.

Visual inspection (Figure 3) suggested a positive association between L2 proficiency and Gm performance. To explore this trend further, we conducted exploratory trend analyses using the *emmeans* package (Lenth 2023), estimating slopes within each group and sentence type. Among autistic children, higher L2 proficiency significantly predicted better performance on Gm sentences (Estimate = 0.373, SE = 0.158, 95% CI [0.064, 0.683]), while no such effect was found in NT children (Estimate = −0.064, SE = 0.112, 95% CI [−0.283, 0.154]). These findings support visual trends but should be interpreted cautiously due to the nonsignificant higher‐order interaction.

#### 3.2.3 RQ3: Effect of L2 Proficiency on MA Requiring Analyzed Knowledge

The model for RQ3, identical to that used for RQ2, revealed a significant three‐way interaction between *group*, *L2 proficiency*, and *sentence type* (comparison [b], gM vs. GM; Estimate = −0.292, SE = 0.119, *z* = −2.459, *p* = 0.014; Table 4). In NT children, the interaction between *L2 proficiency* and *comparison [b]* was significant (Estimate = 0.201, SE = 0.073, *z* = 2.743, *p* = 0.006; Table A in Appendix S6), with a marginally significant positive effect of L2 proficiency on gM sentences (*p* = 0.058; Table B in S6). No effect of L2 proficiency was found for GM sentences (*p* = 0.918; Table C in S6). In autistic children, the interaction for gM versus GM was not significant (*p* = 0.332; Table D in S6), but higher L2 proficiency significantly predicted better overall task performance (Estimate = 0.331, SE = 0.144, *z* = 2.301, *p* = 0.021).

#### 3.2.4 Common Effects Across Models

Across models for RQ1 and [Link], [Link], a consistent pattern emerged: *age*, *nonverbal IQ*, and *receptive vocabulary* in the testing language were all significant positive predictors of task performance (Tables 3, 4). Performance also varied by testing language: participants tested in English scored significantly below the grand mean, while those tested in French and German scored significantly above it.

## 4 Discussion

This study investigated (1) whether reported bilingualism effects on MA requiring *cognitive control* and *analyzed knowledge* previously observed in two‐dimensional GJTs were replicated in a sample of *N* = 180 NT children aged 5–12; and (2) whether these effects extended to *N* = 90 children diagnosed with ASD. It furthermore clarified the role of L2 proficiency on metalinguistic performance in both populations.

### 4.1 Findings and Implications

#### 4.1.1 Effects on MA Requiring Cognitive Control: Bilinguals Versus Monolinguals

We first examined whether bilingual NT and autistic children outperformed monolingual peers on sentences requiring *cognitive control* (Gm sentences), controlling for age, nonverbal IQ, and language skills in the testing language. Bilingual autistic children significantly outperformed monolingual autistic peers, consistent with evidence of bilingual advantages in executive functions in autism (Gonzalez‐Barrero and Nadig 2019; Li et al. 2017; Peristeri et al. 2021; Romero and Uddin 2021; Sharaan et al. 2021). While bilingual NT and monolingual NT children performed comparably, bilingual autistic children performed comparably to bilingual NT peers. Among monolinguals, NT children outperformed autistic peers on MA requiring *cognitive control*, consistent with well‐documented executive function challenges in autism (Craig et al. 2016; Demetriou et al. 2018). These patterns suggest that bilingualism may act as a naturalistic compensatory mechanism, reducing performance gaps between autistic and NT children on tasks engaging *cognitive control*. Such compensatory effects align with findings that bilingualism can enhance task monitoring and set‐shifting in autistic individuals (Gonzalez‐Barrero and Nadig 2019; Peristeri et al. 2021; Sharaan et al. 2021).

These findings add to evidence that dual‐language exposure can support, rather than hinder, cognitive and linguistic development in autism (Drysdale et al. 2015; Gilhuber et al. 2023). They suggest that the bilingual advantage in MA seen in NT children (Adesope et al. 2010) may extend to autistic children, and that bilingualism‐related advantages in cognitive control found in autistic individuals (Gonzalez‐Barrero and Nadig 2019; Peristeri et al. 2021) may also generalize to linguistic tasks.

The bilingual advantage observed in autistic children is theoretically consistent with the demands of sentences requiring *cognitive control*, which are grammatically correct but semantically anomalous. To respond accurately, children must inhibit the automatic processing of meaning and focus on grammatical form. Such inhibition and goal maintenance rely on interference control, conflict monitoring, and working memory, which are core components of the executive system (Bialystok and Ryan 1985; Diamond 2013; Hakes 1980). Bilingual experience is thought to strengthen these same processes through continual management of competing linguistic systems (Abutalebi and Green 2016; Costa et al. 2009; Green and Abutalebi 2013; Hilchey and Klein 2011). Accordingly, the advantage observed in bilingual autistic children may reflect enhanced conflict monitoring and flexibility, which are often vulnerable in autism (Craig et al. 2016; Demetriou et al. 2018).

In contrast, sentences requiring *analyzed knowledge* (i.e., meaningful but ungrammatical sentences) rely primarily on explicit understanding of grammatical rules rather than cognitive control. Bilingualism is not typically expected to confer an advantage on such items (Bialystok 1986, 1988; Bialystok and Barac 2012). The absence of group differences on these sentences indicates that, when vocabulary level is controlled, autistic and NT children show similar levels of performance (see Wolfer et al. 2024 for a similar conclusion). This pattern reinforces the view that the observed differences on control‐demanding sentences arise from the *executive control* processes those items engage, rather than from disparities in linguistic knowledge.

Taken together, these results highlight that bilingualism appears to support metalinguistic performance most strongly when tasks engage executive control processes. This aligns with findings indicating that bilingualism may offer particular cognitive support in populations experiencing challenges in executive function (Romero and Uddin 2021). More broadly, bilingual advantages are not global but context‐dependent, emerging when tasks require conflict monitoring or flexibility, or when populations show executive function challenges (Lowe et al. 2021; Paap 2022; Romero and Uddin 2021).

No bilingual advantage was observed in NT children on Gm sentences, diverging from earlier studies (Bialystok 1986, 1988; Bialystok and Majumder 1998). This may reflect differing bilingual experiences: unlike prior samples in immersion programs with sustained dual‐language use (Bialystok 1986, 1988; Cromdal 1999), our participants had varied linguistic backgrounds and were not enrolled in structured programs. Without such immersion, which may heighten cognitive demands of managing two languages (Linck et al. 2009; Nicolay and Poncelet 2013, 2015), advantages in cognitive control may be less pronounced.

Individual differences in executive function may also explain variability. Abilities such as phonological short‐term memory (STM) and working memory (WM), which respectively support storage and manipulation of verbal information (Diamond 2013), are critical in GJT. While some studies report bilingual WM advantages (e.g., Morales et al. 2013), findings are mixed (e.g., Giovannoli et al. 2020; Lowe et al. 2021; Monnier et al. 2022), and these abilities were not directly assessed in this study, leaving their role unclear. Replicating bilingual advantages remains challenging (Bak 2016; Paap 2022), given bilingualism's multidimensional nature (de Bruin 2019; Luk 2015) and unmeasured factors such as language status, exposure, and sociolinguistic context (Luk and Grundy 2023; Masullo et al. 2024). These results support calls to move beyond categorical monolingual‐bilingual comparisons toward continuous measures reflecting individual differences.

One such factor is L2 proficiency. Prior work indicates that only bilingual children with high L2 proficiency outperform monolinguals on tasks requiring *cognitive control* (Cromdal 1999). When tested in their most proficient language, high L2‐proficient bilinguals outperformed their peers with lower L2 proficiency on MA requiring *analyzed knowledge*, suggesting a positive impact of L2 proficiency. However, its effect as a continuous predictor on grammaticality judgments involving both *analyzed knowledge* and *cognitive control* has not been systematically examined. We address this gap in RQ2 and RQ3.

#### 4.1.2 Effects of L2 Proficiency on MA Requiring Cognitive Control

No statistical evidence emerged for an effect of L2 proficiency on Gm sentences in either NT or autistic children. However, post hoc estimated marginal means suggested a positive association between L2 proficiency and performance on *cognitive control* among autistic children. As this effect did not reach significance in the primary model, it should nevertheless be interpreted with caution.

These results suggest that factors beyond L2 proficiency may underlie the enhanced performance of autistic children (see RQ1), and NT bilinguals in previous studies (Bialystok 1986; Bialystok and Majumder 1998; Cromdal 1999). This highlights the multidimensional nature of bilingualism and supports calls to integrate categorical and continuous approaches to capture how diverse bilingual experiences impact cognitive and linguistic outcomes (Kremin and Byers‐Heinlein 2021).

Consistent with earlier claims (Bialystok and Barac 2012), advantages in MA involving cognitive control may relate more to domain‐general effects of bilingualism than to L2 proficiency alone. Future research should identify which aspects of bilingual experience, known to influence cognitive control, predict performance on metalinguistic tasks.

#### 4.1.3 Effects of L2 Proficiency on MA Requiring Analyzed Knowledge

The final research question examined whether *L2 proficiency* predicted performance on sentences requiring *analyzed knowledge*. No significant effects emerged in either group, though a positive trend was observed. This contrasts with earlier group‐based studies (Bialystok 1988; Cromdal 1999), reporting that “fully” bilingual children outperformed “partially” bilingual peers on such items. However, those studies did not assess L2 proficiency as a continuous variable.

In the present study, there was insufficient evidence to support the hypothesis that higher L2 proficiency independently enhances analyzed knowledge. Although managing two linguistic systems may support grammatical rule abstraction (Bialystok 1988; Galambos and Goldin‐Meadow 1990), factors such as richness of input may also contribute to metalinguistic performance.

Notably, autistic children with higher L2 proficiency outperformed peers with lower L2 proficiency on the GJT overall, suggesting that the MA “boost” documented in bilingual NT children (Adesope et al. 2010) may also extend to children with ASD.

#### 4.1.4 Additional Effects

Across all models, age positively predicted performance, consistent with developmental gains in metalinguistic competence over school years (Bialystok 1986; Melogno et al. 2022; Scribner and Cole 1981). Nonverbal IQ also predicted higher scores, aligning with previous work (Wolfer et al. 2024) and reflecting the cognitive demands of the two‐dimensional GJT (see Appendix S7 for supplementary exploratory analyses depicting accuracy on Gm sentences as a function of age, nonverbal IQ, and SCQ scores). Receptive vocabulary in the testing language significantly contributed to performance, supporting evidence of links between vocabulary and metalinguistic ability (Nagy 2007; Smith and Tager‐Flusberg 1982).

Although receptive vocabulary and nonverbal IQ were lower in the autistic group than in neurotypical peers, both factors were statistically controlled in all models. Therefore, group differences in metalinguistic awareness likely reflect task‐specific processes rather than general cognitive or language‐level disparities. Nonetheless, because language proficiency and reasoning abilities contribute to metalinguistic performance, these findings highlight the importance of considering individual variability in future research.

Beyond participant‐level predictors, performance varied by testing language: English‐tested participants scored below the grand mean, whereas participants tested in German and French scored above. Post hoc analyses indicated that German‐tested children were older (109 ± 19 months) and mostly NT (78%), likely contributing to higher scores. French‐tested children were younger (100 ± 23 months) with a balanced distribution of autistic and NT participants (53% autistic), suggesting that other unmeasured factors, such as literacy exposure may have supported performance. English‐tested children were not younger (107 ± 27 months) but included a higher proportion of autistic participants (81%), possibly explaining lower scores. Similar patterns of lower performance for English‐tested participants have been reported with this task (Wolfer et al. 2024), potentially due to language‐specific features of the English stimuli, such as variability in use or omission of the—s agreement marker.

### 4.2 Limitations

Several limitations can be mentioned. First, due to the complexity of the tasks and the need to assess cognitive and linguistic abilities using standardized measures, only children able to sustain attention throughout the assessment completed the full protocol. Although efforts were made to enhance accessibility, the sample may not fully represent the heterogeneity of the autism spectrum. In particular, children with low IQ were not included, reflecting a common limitation in autism research (Russell et al. 2019).

Second, while L2 proficiency was assessed in a detailed manner across four domains (*comprehension*, *speaking*, *reading*, and *writing*), it relied on parent report. Although informative, parent‐reported proficiency may be less reliable than standardized, objective assessments (Bishop and McDonald 2009).

Third, this study did not consider the impact of a third language (L3) on performance, despite growing interest in this area (Cenoz 2003). Future research may explore how L3 exposure influences metalinguistic awareness and cognitive outcomes in both NT and autistic populations.

## 5 Conclusion

This study fills a critical gap by examining how bilingualism influences MA in autism, applying a two‐dimensional GJT framework previously used only with NT children to a large, linguistically diverse sample (90 autistic, 180 NT participants). By integrating both categorical and continuous measures of bilingualism, we provide a more nuanced account of how bilingual experience impacts MA in both populations.

Among NT children, the lack of a bilingual advantage in items requiring *cognitive control* calls into question claims of universal bilingual benefits. In contrast, bilingual autistic children outperformed their monolingual peers on such items, and higher L2 proficiency predicted better overall GJT performance. These findings suggest that bilingualism is not only non‐detrimental but may offer cognitive and linguistic benefits in autism.

This supports growing advocacy for dual‐language maintenance in autistic children (Beauchamp and MacLeod 2017; Drysdale et al. 2015; Lund et al. 2017), and contributes to a more inclusive, theoretically grounded approach to bilingualism research (Kremin and Byers‐Heinlein 2021; Rothman et al. 2023), one that accounts for developmental and individual variability.

## Author Contributions

**Pauline Wolfer:** conceptualization, data curation, formal analysis, investigation, methodology, resources, supervision, visualization, writing – original draft. **Franziska Baumeister:** conceptualization, methodology, data curation, investigation, resources, supervision, writing – review and editing. **Stephanie Durrleman:** conceptualization, methodology, resources, supervision, writing – review and editing, project administration, funding acquisition. All authors reviewed and approved the manuscript for publication.

## Funding

This study is supported by the Swiss National Science Foundation (SNSF) awarded to Stephanie Durrleman (PR00P1_193104/1).

## Ethics Statement

This study was approved by the Swiss Association of Research Ethics Committees *Swissethics* (Project ID‐2022‐00878), the Institutional Review Board of the University of Connecticut (US), the Research Ethics Board Office at McGill University in Montreal (Canada), the Psychology Research Ethics Committee of the University of Edinburgh (UK), the Institutional Review Board of Emerson College Boston (US), and the Research Ethics Committee of the Unio Catalana Hospitals Foundation (Spain). All work has been carried out in accordance with The World Medical Association's Declaration of Helsinki for experiments involving humans.

## Consent

All parents provided informed written consent for their child's participation prior to their inclusion in the study.

## Conflicts of Interest

The authors declare no conflicts of interest.

## Supporting information


**Appendix S1:** Supporting information.
**Appendix S1:** Bilingual characteristics of the sample.
**Appendix S1C:** L2 proficiency calculation.
**Appendix S2:**. Examples of GJT items in the different languages.
**Appendix S3:** References of the different language versions of the peabody picture vocabulary test (PPVT‐4).
**Appendix S4:** Detailed models specifications.
**Appendix S5:** Post hoc investigations of interactions for RQ1.
**Appendix S6:** Post hoc investigations of interactions for RQ2/RQ3.
**Appendix S7:** Supplementary exploratory descriptive analyses (monolinguals, Gm sentences).

## Data Availability

The data and analysis scripts are openly available at: https://osf.io/kbyc6/ [DOI 10.17605/OSF.IO/KBYC6].
