# Supplementary material for: Bilingualism Enhances Metalinguistic Awareness in Autism: Extending the Two‐Dimensional Grammaticality Judgment Task
Source: Autism Res. 2025 Dec 29;19(3):e70173. doi: 10.1002/aur.70173 (PMC12996853; doi:10.1002/aur.70173)
Supplement: Supplementary file 1 — Appendix S1: Supporting information. Appendix S1: Bilingual characteristics of the sample. Appendix S1C: L2 proficiency calculation. Appendix S2:. Examples of GJT items in the different languages. Appendix S3: References of the different language versions of the peabody picture vocabulary test (PPVT‐4). Appendix S4: Detailed models specifications. Appendix S5: Post hoc investigations of interactions for RQ1. Appendix S6: Post hoc investigations of interactions for RQ2/RQ3. Appendix S7: Supplementary exploratory descriptive analyses (monolinguals, Gm sentences). [file AUR-19-0-s001.docx]

**Supplemental Material - Appendix**

**Appendix S1**. **Bilingual characteristics of the sample**

- *Table A. Variety of Languages participants were exposed to.*


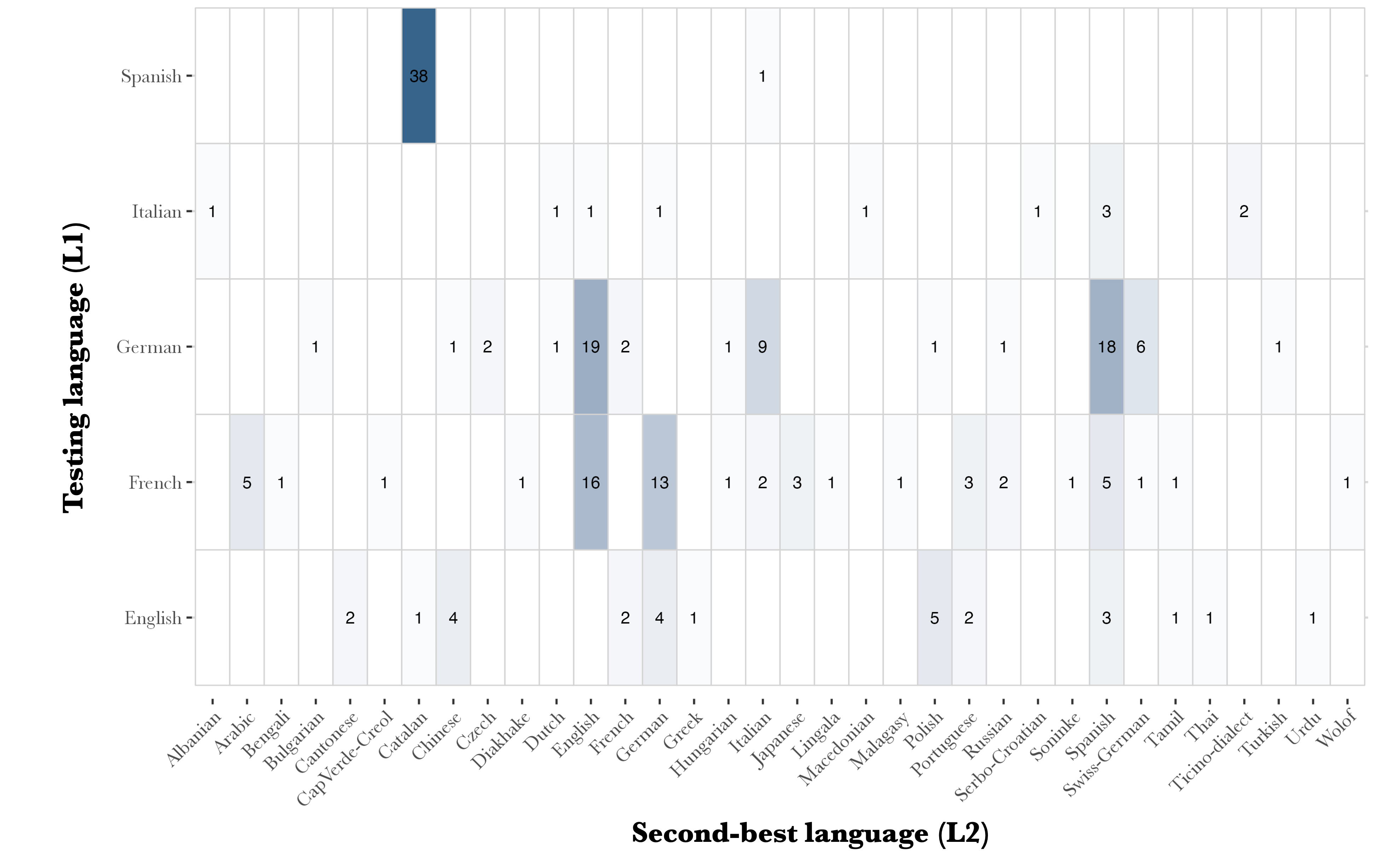


Numbers represent number of participants.

- *Table B. Description of bilingual-related characteristics of the sample.*

|  | **Autistic children (N=90)** | | | | | | | | | | **Neurotypical children (N=180)** | | | | | | | | |  |  |
| --- | --- | --- | --- | --- | --- | --- | --- | --- | --- | --- | --- | --- | --- | --- | --- | --- | --- | --- | --- | --- | --- |
|  | **L1** | | | **L2** | | | **L3** | | | | **L1** | | | | **L2** | | | | **L3** |  |  |
| **Age of First Exposition**  Mean (SD) [range] | \| 8.17 (18.1) \| \| --- \| \| 0 [0, 95] \| | \| 14.2 (25.9) \| \| --- \| \| 0 [0, 120] \| | | | \| 26.1 (26) \| \| --- \| \| 24.5 [0, 84] \| | | | \| 5.25 (12.9) \| \| --- \| \| 0 [0, 60] \| | | | | \| 10.1 (22.3) \| \| --- \| \| 0 [0, 97] \| | | | | \| 25.8 (37.2) \| \| --- \| \| 1.00 [0, 126] \| | | | |  |  |
| **Place of First Exposition** | | | | | | | | | | | | | | | | | | | |  |  |
| Home | 73 (81.1%) | 49 (54.4%) | | | 8 (8.9%) | | | | 138 (76.7%) | | | | 87 (48.3%) | | | | 19 (10.6%) | | | |  |
| Other | 5 (5.6%) | 5 (5.6%) | | | 1 (1.1%) | | | | 9 (5.0%) | | | | 12 (6.7%) | | | | 4 (2.2%) | | | |  |
| School | 12 (13.3%) | 17 (18.9%) | | | 13 (14.4%) | | | | 33 (18.3%) | | | | 29 (16.1%) | | | | 17 (9.4%) | | | |  |
| **Relative exposure since birth (%)** | \| 72.2 (29.0) \| \| --- \| \| 81 [0, 100] \| | | \| 31.4 (24.9) \| \| --- \| \| 30.1 [0, 92.6] \| | | | \|  \| \| --- \| \| 12.3 (14.0) \| \| 6.6 [0, 45] \| | | | | \| 72.0 (27.8) \| \| --- \| \| 76.2 [4.19, 100] \| | | | | \| 34.8 (23.9) \| \| --- \| \| 35.9 [0, 95.8] \| | | | | \| 14.7 (14.7) \| \| --- \| \| 9.44[0, 50] \| | | | |
| **Relative use in the last year (%)** | \| 82.1 (22.2) \| \| --- \| \| 91 [15, 100] \| | \| 21.2 (21.5) \| \| --- \| \| 14 [0, 83] \| | | | \| 4.55 (10.0) \| \| --- \| \| 1.50 [0, 47] \| | | | | \| 78.4 (25.4) \| \| --- \| \| 91.0 [6, 100] \| | | | | \| 27.8 (24.3) \| \| --- \| \| 23 [0, 94] \| | | | | \| 8.05 (9.94) \| \| --- \| \| 3 [0, 40] \| | | | |  |

Note. In the group of autistic children, *N* = 71 caregivers reported the presence of a second Language (L2), and *N* = 22 the presence of a third Language (L3). In the group of neurotypical children, *N* = 128 caregivers reported the presence of a L2 and *N* = 40 of a L3.

- *Appendix S1C. L2 proficiency calculation*

To index participant’s L2 proficiency for RQ2 and RQ3, we selected the parents’ estimate of their child’s proficiency in their second-best language L2. Specifically, parents indicated on a 4-point Likert-scale the level of proficiency of their child in *speaking*, *understanding*, *writing* and *reading* any language to which the child was exposed. Scores on these subindices were averaged per language. The result was a percentage indicating the proficiency in that language, from 0% (no proficiency in that language) to 100% (excellent proficiency). Hence, the percentage corresponding to the second-best language other than the language of testing (L1) indicated L2 proficiency. For participants whose parents reported no L2 (i.e., ‘true’ monolinguals), L2 proficiency was set to 0.

**Appendix S2.** **Examples of GJT items in the different languages**

|  | GM | gM  *Analyzed knowledge* | Gm  *Cognitive control* | gm |
| --- | --- | --- | --- | --- |
|  | Grammatically correct,  Semantically appropriate | Grammatically incorrect, Semantically appropriate | Grammatically correct,  Semantically  odd | grammatically incorrect, Semantically  odd |
| ENGLISH | Mark goes to the swimming pool with a friend. | Tonight there are clouds and stars in sky *the*. | The *broom* wakes up every morning. | The rain *climb* tonight in the sandbox. |
| FRENCH | Marc va à la piscine avec un ami. | La nuit il y a nuages *des* dans le ciel. | Le *balai* se réveille tous les matins. | La pluie *grimpons* ce soir dans le caillou. |
| GERMAN | Mark geht mit einem Freund ins Schwimmbad. | Es gibt in Nacht *der* Wolken am Himmel. | Der *Besen* wacht jeden Morgen auf. | Der Regen *klettern* heute Nacht in den Stein. |
| ITALIAN | Marco va al mare con un amico. | Di notte sono stelle *ci* in cielo. | La *scopa* si sveglia la mattina. | La pioggia si *arrampicare* sul sasso |
| SPANISH | Marcos va a la piscina con amigos. | Esta noche hay nubes en cielo el. | La escoba se levanta cada día. | La lluvia subir ayer en el castillo. |

**Appendix S3. References of the different language versions of the Peabody Picture Vocabulary Test (PPVT-4).**

To assess receptive vocabulary in the children’s L1, the respective versions of the *Peabody Picture Vocabulary Test* (PPVT-4) have been proposed:

- **In English**: Dunn, L. M., & Dunn, D. M. (2007). PPVT-4: Peabody picture vocabulary test. Pearson Assessments.
- **In French**: Dunn, L. M., Dunn, L. M., & Thériault-Whalen, C. M. (1993). Echelle de vocabulaire en images Peabody: EVIP. PSYCAN.
- **In German**: Lenhard, A., Lenhard, W., Segerer, R., & Suggate, S. (2015). Peabody picture vocabulary test-4. Deutsche Fassung. (Pearson Assessment).
- **In Italian**: Stella, G., Pizzoli, C., & Tressoldi, P. (2000). Il Peabody Test—Test di vocabolario ricettivo. Omega Edizione, Torino.
- **In Spanish**: Dunn, L. M., Dunn, L. M., & Arribas Aguila, D. (2006). PPVT-III Peabody test de vocabulario en imágenes. TEA Ediciones.

**Appendix S4**. **Detailed models specifications**

Generalized mixed-effects models with a logit-link function were fitted independantly for each research question, using the *glmer* function from the *lme4 package* (Bates et al., 2015) in *R* (version 2024.04.2+764; R Core Team, 2020). A separate model was created for each RQ, with performance at the item level (0 or 1) as the dependent variable.

Two planned sentence-type contrasts were entered as contrast-coded predictors:

**[a] Gm vs. GM sentences**, indexing the *cognitive control* dimension, and

**[b] gM vs. GM sentences**, indexing the *analyzed knowledge* dimension.

For RQ1, we included the main effects and full three-way interactions between *diagnostic group* (ASD/NT, sum-coded), *bilingual status* (monolingual/bilingual, sum-coded) and *sentence type.* For RQ2 and RQ3, we models included the main effects and full three-way interactions between *diagnostic* *group* (ASD/NT, sum-coded), *L2 proficiency* (scaled proficiency in the L2) and *sentence type*.

All models included the following covariates as fixed effects: *age* (scaled), *language of testing* (sum-coded), *non-verbal IQ* (scaled), and *receptive vocabulary* (z-score), given their known influence on GJT performance (Bialystok, 1986; Tremblay, 2005; Wolfer et al., 2024).

Participants and items were included as random intercepts. All continuous predictors were scaled, and model diagnostics confirmed no problematic multicollinearity (VIF ≤ 5). Significant interactions were resolved hierarchically.

**Cited references**

Bates, D., Mächler, M., Bolker, B., & Walker, S. (2015). Fitting linear mixed-effects models using lme4. *Journal of Statistical Software*, *67*(1), 1–48. <https://doi.org/10.18637/jss.v067.i01>

Bialystok, E. (1986). Factors in the growth of linguistic awareness. *Child Development*, 498–510.

R Core Team. (2020). *R Core Team (2020). A language and environment for statistical computing.* (Vienna, Austria).

Tremblay, A. (2005). Theoretical and methodological perspectives on the use of grammaticality judgment tasks in linguistic theory. *Sec. Lang. Stud.*, *24*.

Wolfer, P., Baumeister, F., Rudelli, N., Corrigan, G., Naigles, L. R., & Durrleman, S. (2024). Exploring metalinguistic awareness in school-aged autistic children: Insights from grammatical judgment. *Journal of Autism and Developmental Disorders*. <https://doi.org/10.1007/s10803-024-06569-y>

**Appendix S5**. **Post-hoc investigations of interactions for RQ1**

*Table A*. Output of the GLMM for RQ1 in NT children only

|  | Est. | Std. Error | z value | *p* |
| --- | --- | --- | --- | --- |
| (Intercept) | 2.249 | 0.160 | 14.062 | < .001 |
| Bilingual status | -0.027 | 0.203 | -0.135 | .893 |
| Gm vs. GM [a] | -0.372 | 0.190 | -1.958 | .050 |
| gM vs. GM [b] | -1.357 | 0.187 | -7.266 | < .001 |
| Sentence type [omnibus] | 0.156 | 0.229 | 0.681 | .496 |
| Age | 0.927 | 0.089 | 10.367 | < .001 |
| Non-verbal IQ | 0.229 | 0.099 | 2.313 | .021 |
| Receptive Vocabulary in L1 (PPVT) | 0.238 | 0.081 | 2.933 | .003 |
| Language_English | -2.370 | 0.819 | -2.894 | .004 |
| Language_French | 3.008 | 0.881 | 3.412 | .001 |
| Language_German | 0.546 | 0.786 | 0.695 | .487 |
| Language_Italian | -1.073 | 1.103 | -0.972 | .331 |
| Bilingual status:Gm vs. GM [a] | -0.302 | 0.166 | -1.823 | .068 |
| Bilingual status:gM vs. GM [b] | 0.549 | 0.163 | 3.359 | .001 |
| Bilingual status:Sentence type [omnibus] | -0.286 | 0.193 | -1.481 | .139 |

*Table B*. Output of the GLMM for RQ1 in autistic children only

|  | Est. | Std. Error | z value | *p* |
| --- | --- | --- | --- | --- |
| (Intercept) | 1.533 | 0.183 | 8.364 | < .001 |
| Bilingual status | 0.663 | 0.264 | 2.509 | .012 |
| Gm vs. GM [a] | -0.725 | 0.172 | -4.220 | < .001 |
| gM vs. GM [b] | -0.870 | 0.171 | -5.086 | < .001 |
| Sentence type [omnibus] | -0.046 | 0.210 | -0.220 | .826 |
| Age | 0.686 | 0.122 | 5.637 | < .001 |
| Non-verbal IQ | 0.077 | 0.118 | 0.654 | .513 |
| Receptive Vocabulary in L1 (PPVT) | 0.400 | 0.078 | 5.150 | < .001 |
| Language_English | -4.517 | 1.528 | -2.956 | .003 |
| Language_French | 2.045 | 1.000 | 2.044 | .041 |
| Language_German | 4.343 | 1.292 | 3.362 | .001 |
| Language_Italian | 0.063 | 2.153 | 0.029 | .977 |
| Bilingual status:Gm vs. GM [a] | 0.533 | 0.187 | 2.854 | .004 |
| Bilingual status:gM vs. GM [b] | -0.532 | 0.187 | -2.837 | .005 |
| Bilingual status:Sentence type [omnibus] | 0.299 | 0.228 | 1.312 | .190 |

*Table C*. Output of the GLMM for RQ1 in autistic children only, in Gm sentences specifically

|  | Est. | Std. Error | z value | *p* |
| --- | --- | --- | --- | --- |
| (Intercept) | 1.250 | 0.414 | 3.015 | .003 |
| Bilingual status | 1.755 | 0.638 | 2.753 | .006 |
| Age | 1.409 | 0.322 | 4.384 | < .001 |
| Non-verbal IQ | 0.670 | 0.309 | 2.166 | .030 |
| Receptive Vocabulary in L1 (PPVT) | 0.433 | 0.190 | 2.280 | .023 |
| Language_English | -6.593 | 3.756 | -1.755 | .079 |
| Language_French | 4.067 | 2.413 | 1.685 | .092 |
| Language_German | 5.160 | 3.136 | 1.645 | .100 |
| Language_Italian | 0.258 | 4.831 | 0.053 | .957 |

*Table D*. Output of the GLMM for RQ1 in autistic children only, in GM sentences specifically

|  | Est. | Std. Error | z value | *p* |
| --- | --- | --- | --- | --- |
| (Intercept) | 2.826 | 0.320 | 8.842 | < .001 |
| Bilingual status | 1.195 | 0.491 | 2.435 | .015 |
| Age | 0.579 | 0.226 | 2.565 | .010 |
| Non-verbal IQ | 0.080 | 0.196 | 0.407 | .684 |
| Receptive Vocabulary in L1 (PPVT) | 0.478 | 0.129 | 3.695 | < .001 |
| Language_English | -2.320 | 2.454 | -0.945 | .345 |
| Language_French | 4.687 | 1.743 | 2.689 | .007 |
| Language_German | 2.887 | 2.481 | 1.164 | .244 |
| Language_Italian | -3.505 | 2.878 | -1.218 | .223 |

*Table E*. Output of the GLMM for RQ1 in bilingual children (ASD and NT)

|  | Est. | Std. Error | z value | *p* |
| --- | --- | --- | --- | --- |
| (Intercept) | 1.990 | 0.141 | 14.110 | < .001 |
| Group | -0.277 | 0.202 | -1.373 | .170 |
| Gm vs. GM [a] | -0.485 | 0.172 | -2.827 | .005 |
| gM vs. GM [b] | -1.095 | 0.171 | -6.407 | < .001 |
| Sentence type [omnibus] | 0.025 | 0.210 | 0.120 | .904 |
| Age | 0.824 | 0.085 | 9.732 | < .001 |
| Non-verbal IQ | 0.203 | 0.093 | 2.180 | .029 |
| Receptive Vocabulary in L1 (PPVT) | 0.341 | 0.069 | 4.967 | < .001 |
| Language_English | -2.221 | 0.947 | -2.346 | .019 |
| Language_French | 2.329 | 0.749 | 3.111 | .002 |
| Language_German | 1.894 | 0.799 | 2.371 | .018 |
| Language_Italian | -0.836 | 1.301 | -0.642 | .521 |
| Group:Gm vs. GM [a] | 0.075 | 0.134 | 0.560 | .576 |
| Group:gM vs. GM [b] | -0.108 | 0.134 | -0.808 | .419 |
| Group:Sentence type [omnibus] | 0.118 | 0.162 | 0.730 | .466 |

*Table F*. Output of the GLMM for RQ1 in monolingual children (both ASD and NT)

|  | Est. | Std. Error | z value | *p* |
| --- | --- | --- | --- | --- |
| (Intercept) | 0.891 | 0.264 | 3.370 | .001 |
| Group | -1.231 | 0.352 | -3.497 | < .001 |
| Gm vs. GM [a] | -0.614 | 0.191 | -3.219 | .001 |
| gM vs. GM [b] | -1.139 | 0.185 | -6.175 | < .001 |
| Sentence type [omnibus] | 0.069 | 0.228 | 0.301 | .764 |
| Age | 0.910 | 0.151 | 6.037 | < .001 |
| Non-verbal IQ | 0.129 | 0.139 | 0.930 | .352 |
| Receptive Vocabulary in L1 (PPVT) | 0.257 | 0.100 | 2.564 | .010 |
| Language_French | 1.480 | 0.378 | 3.916 | < .001 |
| Language_German | 0.973 | 0.357 | 2.724 | .006 |
| Language_Italian | 0.465 | 0.437 | 1.064 | .287 |
| Group:Gm vs. GM [a] | -0.768 | 0.211 | -3.644 | < .001 |
| Group:gM vs. GM [b] | 0.983 | 0.210 | 4.677 | < .001 |
| Group:Sentence type [omnibus] | -0.428 | 0.252 | -1.697 | .090 |

*Table G*. Output of the GLMM for RQ1 in monolinguals for Gm sentences

|  | Est. | Std. Error | z value | *p* |
| --- | --- | --- | --- | --- |
| (Intercept) | 2.433 | 0.822 | 2.961 | .003 |
| Group | -3.126 | 1.039 | -3.010 | .003 |
| Age | 1.695 | 0.474 | 3.574 | < .001 |
| Non-verbal IQ | 0.832 | 0.431 | 1.927 | .054 |
| Receptive Vocabulary in L1 (PPVT) | 0.501 | 0.289 | 1.732 | .083 |
| Language_French | 0.285 | 1.067 | 0.267 | .789 |
| Language_German | -1.223 | 1.097 | -1.115 | .265 |
| Language_Italian | -1.798 | 1.246 | -1.442 | .149 |

*To* *address convergence issues, the random-effect structure for this model was simplified to only include random intercepts for participants (1|participant).*

*Table H.* Output of the GLMM for RQ1 in monolinguals for GM sentences

|  | Est. | Std. Error | z value | *p* |
| --- | --- | --- | --- | --- |
| (Intercept) | 2.939 | 0.480 | 6.120 | < .001 |
| Group | -0.727 | 0.573 | -1.268 | .205 |
| Age | 0.142 | 0.244 | 0.582 | .560 |
| Non-verbal IQ | 0.323 | 0.250 | 1.292 | .196 |
| Receptive Vocabulary in L1 (PPVT) | -0.004 | 0.159 | -0.025 | .980 |
| Language_French | 0.276 | 0.600 | 0.459 | .646 |
| Language_German | 0.563 | 0.669 | 0.842 | .400 |
| Language_Italian | -0.228 | 0.669 | -0.341 | .733 |

*To* *address convergence issues, the random-effect structure for this model was simplified to only include random intercepts for participants (1|participant).*

**Appendix S6**. **Post-hoc investigations of interactions for RQ2/RQ3**

*Table A*. Output of the GLMM for RQ2/RQ3, in NT children only

|  | Est. | Std. Error | z value | *p* |
| --- | --- | --- | --- | --- |
| (Intercept) | 2.231 | 0.154 | 14.521 | < .001 |
| L2 proficiency | 0.064 | 0.099 | 0.651 | .515 |
| Gm vs. GM [a] | -0.424 | 0.187 | -2.269 | .023 |
| gM vs. GM [b] | -1.254 | 0.184 | -6.817 | < .001 |
| Sentence type [omnibus] | 0.101 | 0.227 | 0.445 | .656 |
| Age | 0.917 | 0.090 | 10.229 | < .001 |
| Non-verbal IQ | 0.223 | 0.099 | 2.259 | .024 |
| Receptive Vocabulary in L1 (PPVT) | 0.243 | 0.081 | 3.004 | .003 |
| Language_English | -2.227 | 0.834 | -2.670 | .008 |
| Language_French | 2.973 | 0.876 | 3.393 | .001 |
| Language_German | 0.623 | 0.788 | 0.791 | .429 |
| Language_Italian | -0.873 | 1.109 | -0.787 | .431 |
| L2 proficiency: Gm vs. GM [a] | -0.154 | 0.074 | -2.079 | .038 |
| L2 proficiency: gM vs. GM [b] | 0.201 | 0.073 | 2.743 | .006 |
| L2 proficiency: Sentence type [omnibus] | -0.098 | 0.089 | -1.098 | .272 |

*Table B*. Output of the GLMM for RQ2/RQ3, in NT children only, in gM sentences

|  | Est. | Std. Error | z value | *p* |
| --- | --- | --- | --- | --- |
| (Intercept) | 1.192 | 0.368 | 3.236 | .001 |
| L2 proficiency | 0.308 | 0.162 | 1.894 | .058 |
| Age | 1.213 | 0.160 | 7.598 | < .001 |
| Non-verbal IQ | 0.075 | 0.168 | 0.444 | .657 |
| Receptive Vocabulary in L1 (PPVT) | 0.378 | 0.138 | 2.731 | .006 |
| Language_English | -4.884 | 1.439 | -3.394 | .001 |
| Language_French | 4.986 | 1.516 | 3.289 | .001 |
| Language_German | 1.855 | 1.358 | 1.366 | .172 |
| Language_Italian | -0.394 | 1.979 | -0.199 | .842 |

*Table C*. Output of the GLMM for RQ2/RQ3, in NT children only, in GM sentences

|  | Est. | Std. Error | z value | *p* |
| --- | --- | --- | --- | --- |
| (Intercept) | 3.663 | 0.350 | 10.480 | < .001 |
| L2 proficiency | -0.018 | 0.177 | -0.103 | .918 |
| Age | 0.343 | 0.159 | 2.157 | .031 |
| Non-verbal IQ | 0.559 | 0.189 | 2.965 | .003 |
| Receptive Vocabulary in L1 (PPVT) | -0.043 | 0.146 | -0.292 | .770 |
| Language_English | 0.247 | 1.707 | 0.144 | .885 |
| Language_French | 2.632 | 1.964 | 1.340 | .180 |
| Language_German | -3.165 | 1.434 | -2.207 | .027 |
| Language_Italian | -2.870 | 1.727 | -1.662 | .096 |

*Table D.*  Output of the GLMM for RQ2/RQ3, in autistic children only

|  | Est. | Std. Error | z value | *p* |
| --- | --- | --- | --- | --- |
| (Intercept) | 1.736 | 0.184 | 9.436 | < .001 |
| L2 proficiency | 0.331 | 0.144 | 2.301 | .021 |
| Gm vs. GM [a] | -0.590 | 0.166 | -3.563 | < .001 |
| gM vs. GM [b] | -1.003 | 0.165 | -6.068 | < .001 |
| Sentence type [omnibus] | 0.031 | 0.202 | 0.154 | .878 |
| Age | 0.628 | 0.122 | 5.133 | .000 |
| Non-verbal IQ | 0.065 | 0.120 | 0.544 | .587 |
| Receptive Vocabulary in L1 (PPVT) | 0.370 | 0.077 | 4.806 | < .001 |
| Language_English | -4.705 | 1.516 | -3.104 | .002 |
| Language_French | 2.035 | 1.003 | 2.030 | .042 |
| Language_German | 4.334 | 1.295 | 3.348 | .001 |
| Language_Italian | 0.855 | 2.179 | 0.392 | .695 |
| L2 proficiency: Gm vs. GM [a] | 0.087 | 0.094 | 0.924 | .356 |
| L2 proficiency: gM vs. GM [b] | -0.091 | 0.093 | -0.970 | .332 |
| L2 proficiency: Sentence type [omnibus] | 0.039 | 0.113 | 0.342 | .733 |

**Appendix S7**. **Supplementary exploratory descriptive analyses (monolinguals, Gm sentences)**

We conducted exploratory descriptive analyses to examine whether the difference between monolingual autistic and monolingual NT children on sentences requiring greater *cognitive control* (Gm sentences) varied with age, non-verbal IQ, or autistic traits (SCQ). These analyses were intended for illustrative purposes only and were not used for inference; all conclusions rely on the GLMER models reported in the manuscript.

- **Age (Fig. S7.1**) – Visual inspection showed that accuracy on sentences requiring *cognitive control* increased with age in both groups, reflecting general developmental gains in performance. The group difference was evident across the full age range, though slightly narrower at older ages, consistent with the idea that developmental progress may reduce group disparities over time.

**Fig S7.1.**


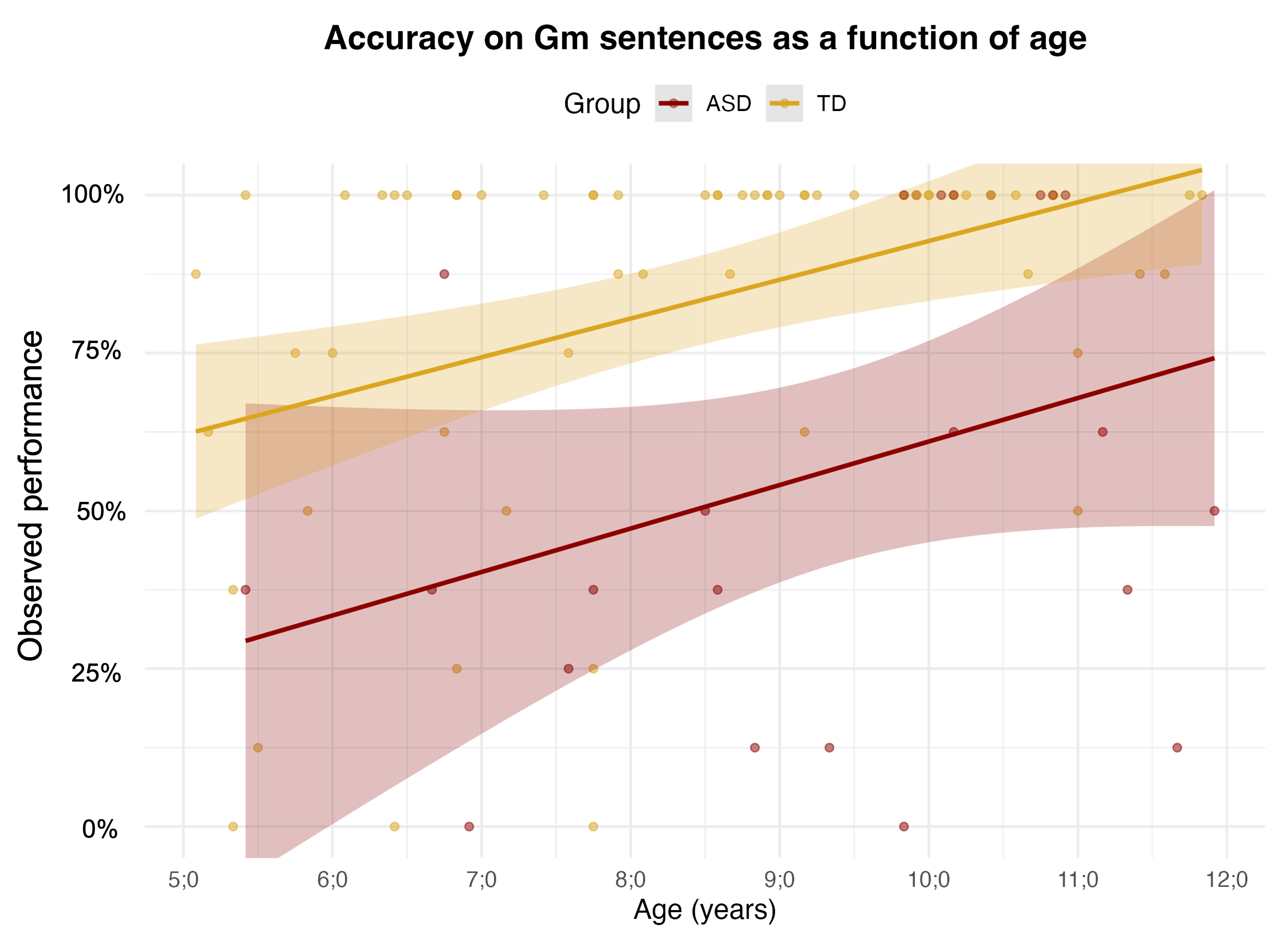


- **Non-verbal IQ (Fig. S7.2)** – Accuracy tended to be higher at greater non-verbal IQ levels in both groups, with the autistic group showing a steeper trend. This pattern aligns with the GLMER finding that non-verbal reasoning contributes to metalinguistic performance, suggesting that stronger reasoning skills may support performance, particularly in autistic children.

**Fig S7.2.**

**
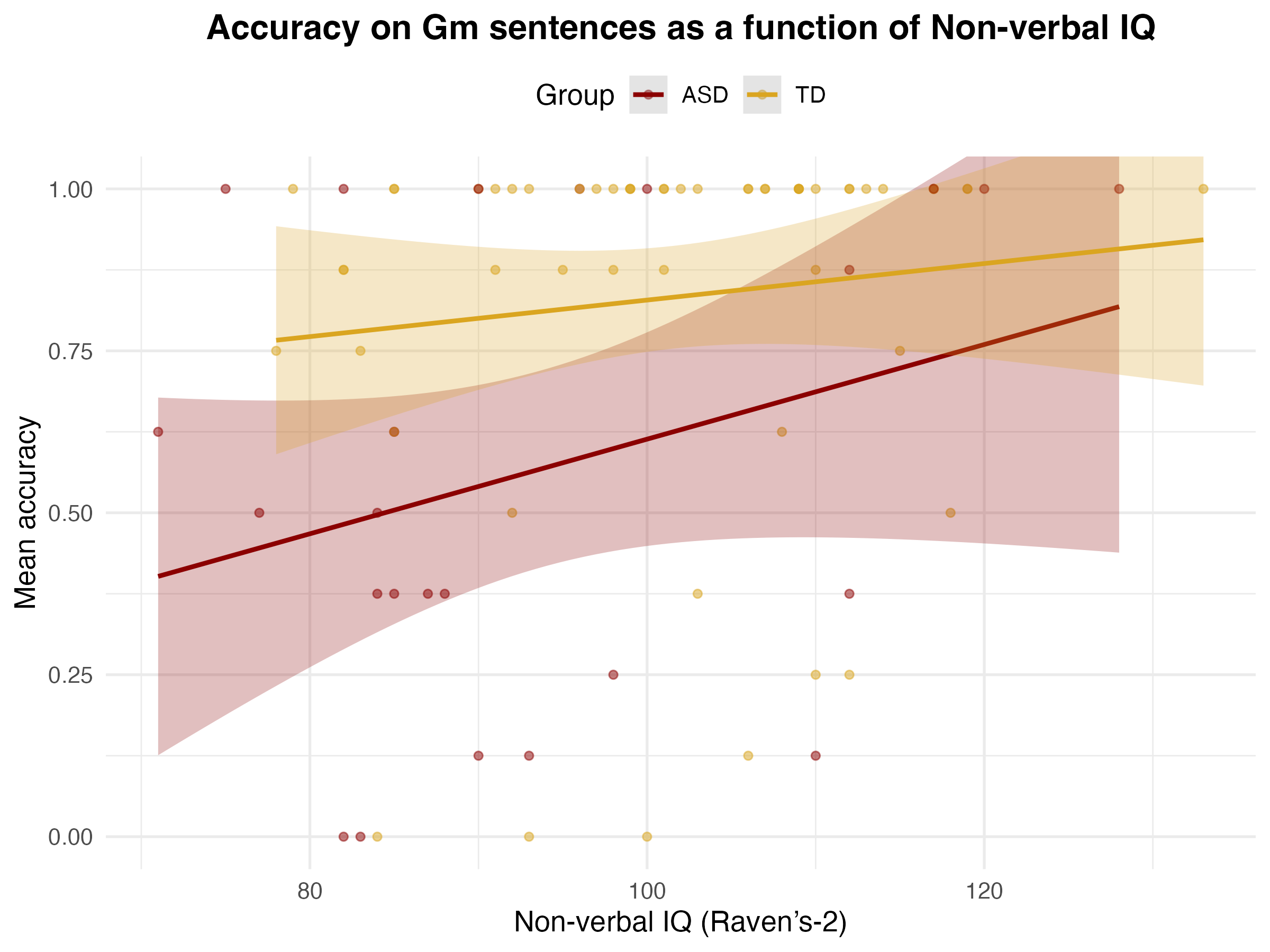
**

- **SCQ total score (Figure S7.3)** – Within the autistic group, a negative trend was observed, suggesting that children with higher SCQ scores tended to show lower accuracy on control-demanding sentences. However, the number of available data points was limited, and confidence intervals were large, indicating that this association should be interpreted with caution.

This exploratory pattern may suggest that greater expression of autistic traits is associated with increased difficulty in tasks requiring *cognitive control*. Future research using clinician-administered measures, such as the ADOS-2 or ADI-R, and larger samples could more precisely assess how specific symptom domains relate to metalinguistic performance.

**Fig. S7.3.**


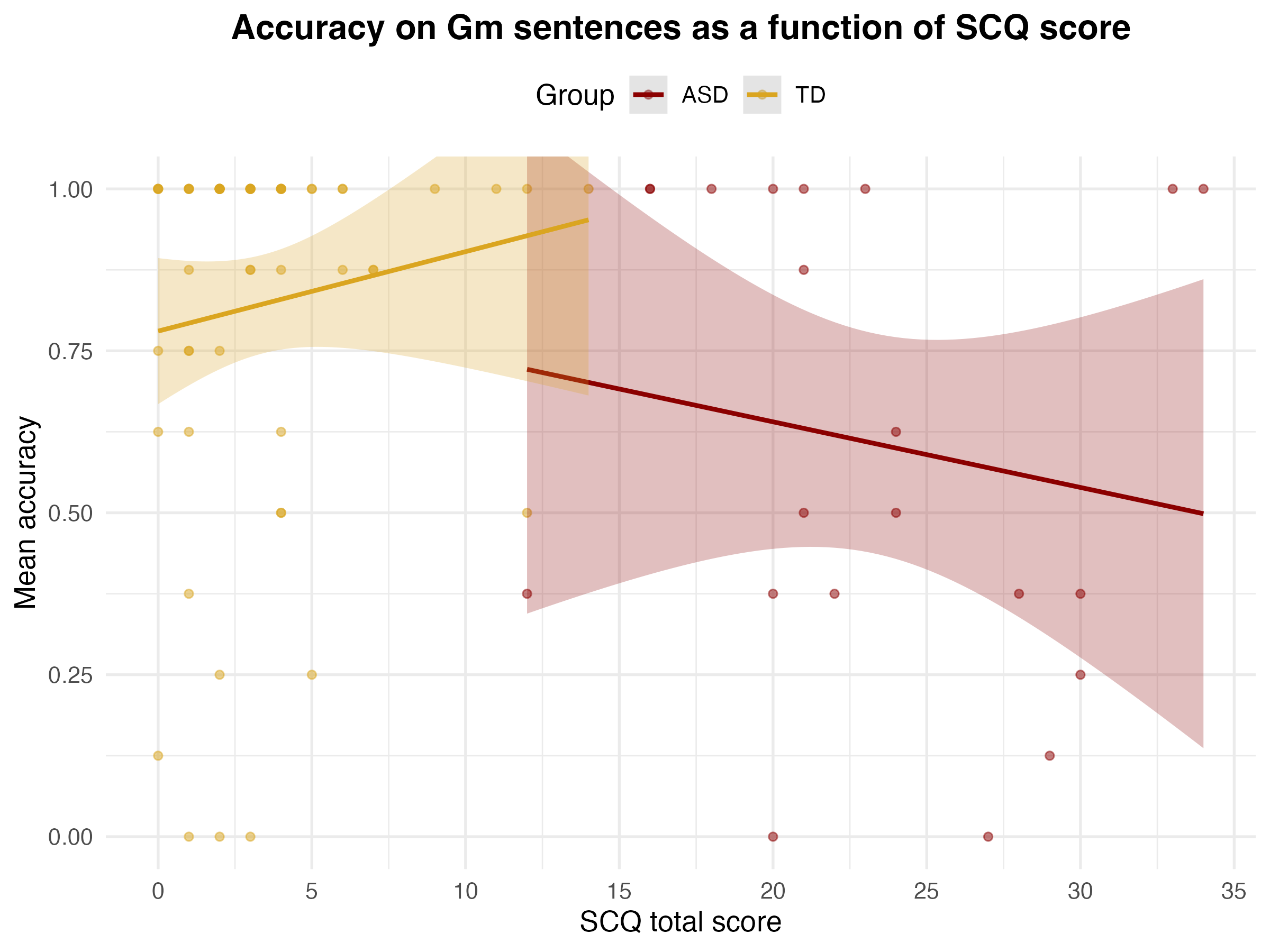


Taken together, these exploratory visualizations suggest that the group difference observed in the GLMER model is robust across a range of ages and cognitive levels, and not driven by a specific subgroup of younger or lower-IQ participants.
